# HDAC and HMT Inhibitors in Combination with Conventional Therapy: A Novel Treatment Option for Acute Promyelocytic Leukemia

**DOI:** 10.1155/2019/6179573

**Published:** 2019-07-18

**Authors:** Aida Vitkevičienė, Giedrė Skiauterytė, Andrius Žučenka, Mindaugas Stoškus, Eglė Gineikienė, Veronika Borutinskaitė, Laimonas Griškevičius, Rūta Navakauskienė

**Affiliations:** ^1^Department of Molecular Cell Biology, Institute of Biochemistry, Life Sciences Center, Vilnius University, Sauletekio Av. 7, LT-01257 Vilnius, Lithuania; ^2^Hematology, Oncology, and Transfusion Medicine Centre, Vilnius University Hospital Santaros Klinikos, Santariskiu Str. 2, LT-08661 Vilnius, Lithuania

## Abstract

Acute promyelocytic leukemia (APL) is characterized by* PML-RARA* translocation, which causes the blockage of promyelocyte differentiation. Conventional treatment with Retinoic acid and chemotherapeutics is quite satisfactory. However, there are still patients who relapse or develop resistance to conventional treatment. To propose new possibilities for acute leukemia treatment, we studied the potential of histone deacetylase (HDAC) inhibitor and histone methyl transferase (HMT) inhibitor to enhance conventional therapy* in vitro* and* ex vivo*. NB4 and HL60 cell lines were used as an* in vitro* model; APL patient bone marrow mononuclear cells were used as an* ex vivo* model. Cell samples were treated with Belinostat (HDAC inhibitor) and 3-Deazaneplanocin A (HMT inhibitor) in combination with conventional treatment (Retinoic acid and Idarubicin). We demonstrated that the combined treatment used in the study had slightly higher effect on cell proliferation inhibition than conventional treatment. Also, enhanced treatment showed stronger effect on induction of apoptosis and on suppression of metabolism. Moreover, the treatment accelerated granulocytic cell differentiation and caused chromatin remodelling (increased H3K14 and H4 acetylation levels).* In vitro* and* ex vivo* models showed similar response to the treatment with different combinations of 3-Deazaneplanocin A, Belinostat, Retinoic acid, and Idarubicin. In conclusion, we suggest that 3-Deazaneplanocin A and Belinostat enhanced conventional acute promyelocytic leukemia treatment and could be considered for further investigations for clinical use.

## 1. Introduction

Acute promyelocytic leukemia (APL) is a subgroup of acute myeloid leukemia, most commonly characterized by chromosomal translocation that generates PML-RAR*α* fusion protein. This protein is responsible for the blockage of promyelocyte differentiation and thus for promyelocyte proliferation and accumulation in the blood [1, 2]. A discovery that all-trans-retinoic acid (RA) targets PML-RAR*α* protein and thereby induces promyelocytic differentiation revolutionized APL treatment. A vast majority of patients achieve complete remission after treatment with various combinations of Retinoic acid with arsenic trioxide and chemotherapeutics [3]. However, a small proportion of APL patients are resistant or develop resistance to RA treatment, which is considered as a critical problem [4].

Therefore, the development of novel treatment strategies is necessary. There is a growing interest in epigenetic therapy. Epigenetic changes such as altered DNA methylation and histone modifications deregulate gene expression and can lead to the induction and maintenance of cancer. Many processes in the cell, for instance, the differentiation blockade and malignant cell proliferation, are influenced by epigenetic alterations [5, 6]. A number of mutated epigenetic modifier genes account for myeloproliferative neoplasms and leukemias [7]. Thus, epigenetic drugs against chromatin regulators are an important tool for cancer treatment [5, 6]. It was demonstrated that, in APL, PML-RAR*α* fusion protein binds DNA and multimerize through its PML domain. Moreover, this aberrant protein recruits various other partners and forms a large protein complex. Among recruited complex proteins, there are various chromatin regulators such as histone deacetylases (HDACs), histone methyltransferases (HMTs), DNA methyltransferases, and polycomb repressive complexes (PRCs) 1 and 2[8]. Thus, targeting not only PML-RAR*α* but also other members of the aberrant complex, such as HDAC and HMT, might potentially improve conventional APL therapy.

HDAC inhibition facilitates chromatin decondensation, which leads to activated gene expression. HDAC inhibitor Belinostat was shown to be effective for relapsed or refractory peripheral T-cell lymphoma treatment in clinical trials. In 2014, it was approved by FDA for this cancer type treatment [9]. There are some widely known HMTs to be involved in carcinogenesis; for example, histone methyl transferase EZH2 is overexpressed in various cancers and it was demonstrated to inhibit acute myeloid leukemia cell differentiation [10]. Epigenetic agent 3-Deazaneplanocin A is an inhibitor of S-adenosyl-L-methionine-dependent HMTs, including EZH2. In preclinical studies, it was shown to inhibit cell proliferation and cause apoptosis in various cancer types [11, 12].

Recently, we showed that epigenetic modifiers 3-Deazaneplanocin A and Belinostat in combination with RA inhibited APL cell proliferation, caused apoptosis, enhanced cell differentiation, and caused chromatin remodelling* in vitro* [13]. Furthermore, in the study with murine xenograft model, we demonstrated that this combined treatment prolonged APL xenograft mice survival and prevented tumour formation [14]. The purpose of this study was to determine the effect of 3-Deazaneplanocin A and Belinostat in combination with conventional treatment (RA + Idarubicin) on NB4 and HL60 cells* in vitro* and on APL patient promyelocytes possessing* PML-RARA* translocation* ex vivo*. We examined the effect of the proposed new epigenetic treatment in combination with conventional treatment on leukemic cell proliferation and granulocytic differentiation potential, changes in oncogene expression, histone modifications involved in chromatin remodelling, and proapoptotic protein expression.

## 2. Materials and Methods

### 2.1. NB4, HL60, and APL Patient Cell Cultivation

NB4 and HL60 cell lines were purchased from DSMZ (Braunschweig, Germany). Bone marrow sample was obtained from a patient diagnosed with APL (promyelocytes consisted of 70% of bone marrow karyocytes;* PML-RARA* translocation was detected). White mononuclear cells were purified from bone marrow aspirate by Ficoll-Paque PLUS density gradient centrifugation (GE Healthcare Chicago, IL, USA). Ethical permission from Vilnius Regional Biomedical Research Ethics Committee (approval no. 158200-16-824-356) and informed consent of the patients were obtained. NB4 cells and freshly purified APL patient cells were seeded at density 0.5 × 10^6^ cells/ml and cultivated in RPMI 1640 medium supplemented with 10% fetal bovine serum, 100 U/ml penicillin, and 100 *μ*g/ml streptomycin (Gibco, Carlsbad, CA, USA) at 37°C in a humidified 5% CO_2_ atmosphere.

### 2.2. Cell Treatment and Proliferation, Survival, Apoptosis, and Cell Cycle Assays

Cell samples were treated with 1 *μ*M Retinoic acid (Sigma-Aldrich, St. Louis, MO, USA), 2 nM or 8 nM Idarubicin (Sigma-Aldrich), 0.5 *μ*M 3-Deazaneplanocin A (Cayman Chemical Company, Ann Arbour, MI, USA), and 0.2 *μ*M Belinostat (PXD101) (Selleckchem, Munich, Germany) in different combinations. Cell proliferation and survival were evaluated by trypan blue exclusion test. Cells were mixed with 0.2% of trypan blue dye (final concentration). Viable and dead (blue coloured) cell numbers were determined by counting the cells in a haemocytometer under the light microscope. For the detection of apoptosis, we used the assay “ApoFlowEx® FITC Kit” (Exbio, Vestec, Czech Republic) according to the manufacturer's instructions. This assay detects viable, early apoptotic, and late apoptotic or necrotic cells according to how they get stained by Annexin V-FITC and Propidium Iodide. Stained cells were analysed on the BD FACS Canto II flow cytometer (Becton Dickinson, Franklin Lakes, NJ, USA). Cell cycle distribution was analysed using standard propidium iodide staining procedure [15].

### 2.3. Mitochondrial Respiration and Glycolytic Activity Measurement

Cell mitochondrial respiration and glycolytic activity were measured using “Agilent Seahorse XF Cell Energy Phenotype Test Kit” (Agilent Dako, Santa Clara, CA, USA) according to the manufacturer's instructions. Plate wells were coated with poly-D-lysine 24 hours before measurement. NB4 cells were seeded at 3 x 10^4^ cells/well, centrifuged for 1 min at 300xg at room temperature, and incubated for 30 min at 37°C without CO_2_. After incubation, cell metabolic phenotype was measured on the Agilent Seahorse XF Extracellular Flux Analyzer (Agilent). Determined oxygen consumption rate (OCR) demonstrates mitochondrial respiration and extracellular acidification rate (ECAR), rate of glycolysis of the cells.

### 2.4. Granulocytic Cell Differentiation Assay

The degree of granulocytic differentiation was evaluated by the ability of cells to reduce soluble nitro blue tetrazolium (NBT) (Sigma-Aldrich) to insoluble blue-black formazan after stimulation with phorbol myristate acetate (PMA) (Sigma-Aldrich). Cells were mixed with 0.1% of NBT and with 100 ng/ml PMA (final concentrations) and were incubated at 37°C for 30 min. Undifferentiated and differentiated (NBT+) cells were counted in a haemocytometer under the light microscope. Differentiated cell percentage was expressed as the NBT^+^ cell number relative to viable cell number.

### 2.5. Gene Expression Analysis by RT-qPCR

Total RNA was purified using TRIzol reagent (Invitrogen, Carlsbad, CA, USA), cDNA was synthesized using SensiFAST™ cDNA Synthesis Kit (Bioline, Memphis, TN, USA), and qPCR was performed using SensiFAST™ SYBR® No-ROX Kit (Bioline) on the RotorGene 6000 system (Corbett Life Science, QIAGEN, Hilden, Germany). Primer sequences (Metabion international AG, Planegg/Steinkirchen, Germany) are presented in Table 1. mRNA levels were normalized to GAPDH expression. Relative gene expression was calculated using ΔΔCt method.

### 2.6. Immunoblotting

Cell lysates were prepared as described previously [15]. Proteins were fractionated in 7.5-15% SDS-PAGE gradient electrophoresis gel and transferred on PVDF membrane. Primary antibodies against WT1 (mouse, clone 6F-H2) (Thermo Fisher Scientific, Waltham, MA, USA), Bcl-2 (mouse, clone 100) (Santa Cruz Biotechnology, Santa Cruz, CA, USA), BAX (rabbit, 2D2) (Santa Cruz Biotechnology), H3K27me3 (rabbit, polyclonal) (Millipore, Billerica, MA, USA), H3K14Ac (rabbit, polyclonal) (Millipore), H4 hyper Ac (rabbit, polyclonal) (Millipore), Caspase-3 (rabbit, clone H-277) (Santa Cruz Biotechnology), GAPDH (mouse, clone 6C5) (Abcam, Cambridge, UK), HRP-conjugated secondary antibodies against mouse immunoglobulins (goat, polyclonal) (Agilent Dako, Santa Clara, CA, USA), and rabbit immunoglobulins (goat, polyclonal) (Agilent Dako) were used according to the manufacturer's instructions. GAPDH was used as loading control. “Clarity Western ECL Substrate” (BIORAD, Hercules, CA, USA) was used for chemiluminescent detection. Signal detection was carried out on ChemiDoc™ XRS+ System (BIORAD). Quantitative evaluation was performed using ImageJ software.

### 2.7. Statistical Analysis

Data are expressed as mean ± standard deviation (S.D.). One-way ANOVA and two-tailed Student's* t*-test were used to calculate the significance of difference between treated and untreated samples; significance was set at P ≤ 0.05 (*∗*).

## 3. Results

### 3.1. Enhanced Treatment Affected APL Cell Proliferation and Death

**Figure 1:**
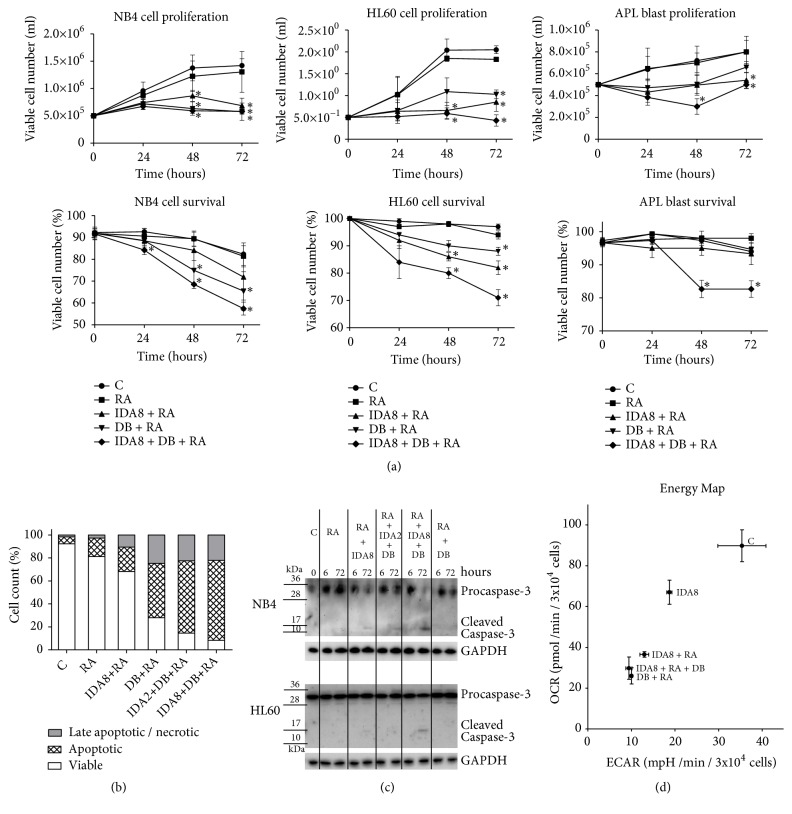
*Cell proliferation and survival after treatment with epigenetic agents in combination with conventional treatment*. NB4 and HL60 cells and APL patient promyelocytes were treated with 1 *μ*M Retinoic acid (RA), 2 nM or 8 nM Idarubicin (IDA2 or IDA8), 0.5 *μ*M 3-Deazaneplanocin A (D), and 0.2 *μ*M Belinostat (B) in different combinations; C: untreated cells. (a) Treated cell proliferation and survival were analysed by trypan blue exclusion test. Results are mean ± S.D. (n = 3); *∗*P ≤ 0.05, calculated by Student's t-test to determine the significance of difference between groups of treated and untreated samples at the same incubation time. (b) Treated NB4 cell apoptosis was evaluated by staining with Annexin V and Propidium Iodide after 72 hours of treatment. (c) Caspase-3 activation was assessed by immunoblot analysis. (d) Metabolic changes in treated NB4 cells were measured using Agilent Seahorse XF Cell Energy Phenotype Test Kit. OCR: oxygen consumption rate (demonstrates cells mitochondrial respiration); ECAR: extracellular acidification rate (demonstrates rate of glycolysis of the cells); results are mean ± S.D. (n = 2).

Bone marrow cells from APL patient possessing* PML-RARA* translocation were purified for white mononuclear cells. NB4 cell line, HL60 cell line, and APL patient white mononuclear cells (70% of blast cells) were treated with 1 *μ*M Retinoic acid, 2 nM or 8 nM Idarubicin, 0.5 *μ*M 3-Deazaneplanocin A, and 0.2 *μ*M Belinostat in different combinations for 72 hours. Drug concentrations were chosen based on previously published work [13] and on unpublished data. Previously, we demonstrated that combined treatment with Belinostat and 3-Deazaneplanocin A had stronger effect on leukemic cells in comparison to their individual effects* in vitro* and* in vivo* [13, 14]. Thus, in this* ex vivo* study, we did not test them separately. In order to compare epigenetic agents 3-Deazaneplanocin A (HMT inhibitor) and Belinostat (HDAC inhibitor) in combination with Idarubicin and Retinoic acid to conventional treatment alone (Idarubicin + Retinoic acid), treated cell proliferation and survival were evaluated every 24 hours (Figure 1(a)). Conventional treatment enhanced with epigenetic agents had slightly higher effect on NB4, HL60, and APL patient cell proliferation and survival compared to treatment with Idarubicin and Retinoic acid alone. However, the combination of 3-Deazaneplanocin A, Belinostat, Idarubicin, and Retinoic acid did not show highly increased cytotoxicity.

In addition, we tested the ability of 3-Deazaneplanocin A and Belinostat combination with Idarubicin and Retinoic acid to induce apoptosis. NB4, HL60, and APL patient cells were treated with different combinations of these agents for 72 hours. Antiapoptotic protein Bcl-2 expression decreased and proapoptotic protein BAX expression increased after the treatment (Figure 4). NB4 cells were stained with Annexin V and Propidium Iodide and analysed (Figure 1(b)). The results show that epigenetic agents 3-Deazaneplanocin A and Belinostat enhanced Idarubicin and Retinoic acid effect; treatment with the combination showed the highest number of apoptotic cells. Moreover, Caspase-3 activation was assessed by immunoblot in NB4 and HL60 cell lines after the treatment (Figure 1(c)). The highest amount of activated (cleaved) Caspase-3 was detected after the treatment with Retinoic acid, 8 nM Idarubicin, 3-Deazaneplanocin A, and Belinostat combination.

Since actively proliferating cells are also active metabolically, we evaluated oxidative phosphorylation and glycolysis rate changes after NB4 cell treatment (Figure 1(d)). Combinations with 3-Deazaneplanocin A and Belinostat had the highest effect on metabolic activity impairment. These findings support proliferation and survival results described above. Taken together, our proposed enhancement of conventional treatment (Idarubicin and Retinoic acid together with epigenetic agents 3-Deazaneplanocin A and Belinostat) had higher effect on cell proliferation and survival inhibition and on apoptosis induction.

### 3.2. Combined Treatment Affected Cell Cycle Progression

**Figure 2:**
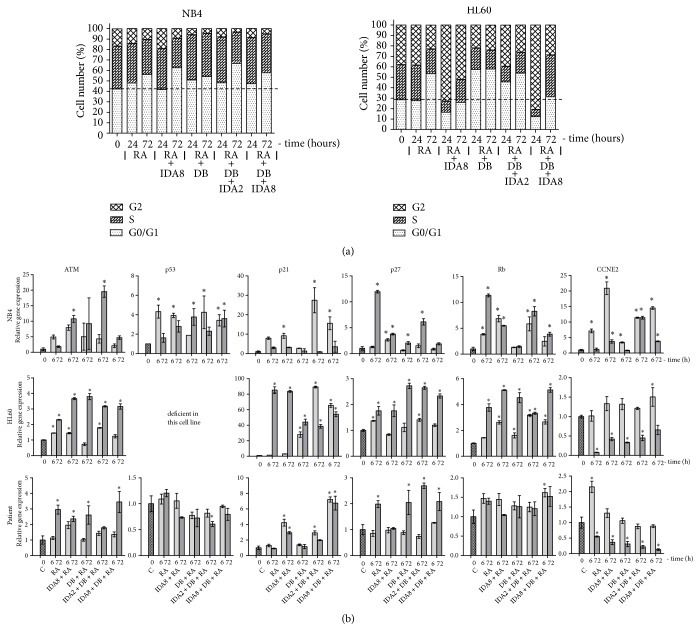
*Cell cycle progression analysis after treatment with epigenetic agents in combination with conventional treatment*. NB4 and HL60 cells and APL patient promyelocytes were treated with 1 *μ*M Retinoic acid (RA), 2 nM or 8 nM Idarubicin (IDA2 or IDA8), 0.5 *μ*M 3-Deazaneplanocin A (D), and 0.2 *μ*M Belinostat (B) in different combinations; C: untreated cells. (a) Cell cycle distribution was analysed using standard propidium iodide method. (b) Cell cycle inhibition related gene expression changes after treatment were measured using RT-qPCR ΔΔCt method. GAPDH was used as a “housekeeping” gene; results are presented as changes in comparison to untreated cells; results are mean ± S.D. (n = 3); *∗*P ≤ 0.05, calculated by one-way ANOVA statistical test.

Cell cycle distribution after NB4 and HL60 cell treatment with Retinoic acid, Idarubicin, 3-Deazaneplanocin A, and Belinostat combinations was evaluated using standard propidium iodide staining procedure (Figure 2(a)). All treatment combinations caused NB4 cell cycle arrest at the phase G0/G1. Retinoic acid, 3-Deazaneplanocin A, and Belinostat combination with 2 nM Idarubicin had slightly higher effect on cell accumulation at the phase G0/G1 compared to the same combination with higher dose of Idarubicin (8 nM). Meanwhile, HL60 cell treatment with higher dose of Idarubicin (8 nM) in combinations for 24 hours caused cell accumulation in G2 cell cycle phase. However, after 72 hours, cell cycle distribution changed and started showing similar tendency as after treatment with other combinations (cell accumulation at the phase G0/G1).

In order to further analyse the proposed combination effect on cell cycle, we assessed cell cycle related gene expression changes by RT-qPCR. Gene expression changes of cell cycle inhibitors* ATM*,* p53*,* p21*,* p27*, and* Rb* and cell cycle activator* CCNE2* (cyclin E2) were analysed in NB4, HL60, and APL patient cells after 6 and 72 hours of treatment with 3-Deazaneplanocin A and Belinostat in different combinations with Retinoic acid and Idarubicin (Figure 2(b)). NB4 cells possess mutated* p53* which is incapable of binding DNA [16]; HL60 cells lack any* p53* expression due to major deletions in the gene [17]. APL patient* p53* mutation status was not tested; however its expression did not increase after the treatment. This demonstrates that cell cycle inhibition in our tested cells might be regulated by p53-independent pathways. Other tested cell cycle inhibitors' genes expression increased in NB4, HL60, and APL patient cells. Cell cycle activator* CCNE2* (cyclin E2) gene expression in HL60 cells after the treatment with 8 nM Idarubicin in combination with Retinoic acid, 3-Deazaneplanocin A, and Belinostat correlated with cell cycle distribution analysis results (Figure 2(a)); initially* CCNE2* expression increased, but, later, after 72 hours, it decreased.

### 3.3. Enhanced Treatment Caused Cell Differentiation

**Figure 3:**
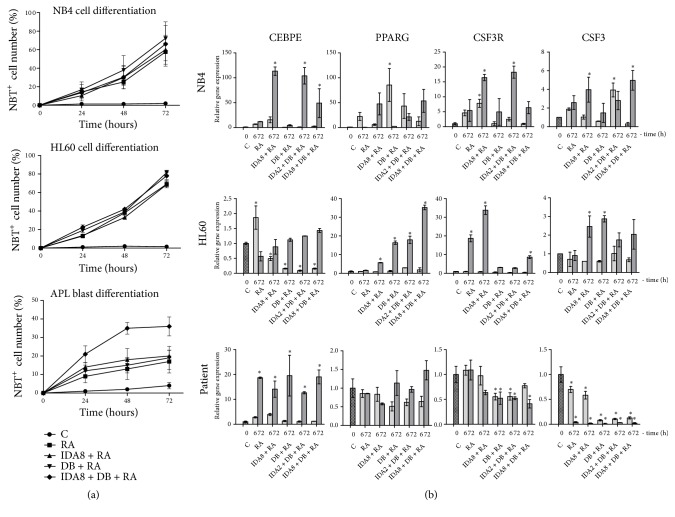
*Granulocytic differentiation potential assessment after treatment with epigenetic agents in combination with conventional treatment*. NB4 and HL60 cells and APL patient promyelocytes were treated with 1 *μ*M Retinoic acid (RA), 2 nM or 8 nM Idarubicin (IDA2 or IDA8), 0.5 *μ*M 3-Deazaneplanocin A (D), and 0.2 *μ*M Belinostat (B) in different combinations for 72 hours; C: untreated cells. (a) Treated cell differentiation was evaluated by nitro blue tetrazolium (NBT) assay. (b) Cell differentiation related gene expression changes after treatment were measured using RT-qPCR ΔΔCt method. GAPDH was used as a “housekeeping” gene; results are presented as changes in comparison to untreated cells; results are mean ± S.D. (n = 3); *∗*P ≤ 0.05, calculated by one-way ANOVA statistical test.

NB4, HL60, and APL patient cells' capacity to differentiate into granulocytes was determined by NBT assay. Cells were treated with new epigenetic modifiers in combination with conventional treatment (3-Deazaneplanocin A and Belinostat combined with Idarubicin and Retinoic acid) and NBT test was performed every 24 hours (Figure 3(a)). NB4 and HL60 cells showed higher capacity to differentiate than APL patient blasts, which could be explained by not as pure APL patient blast population (70% of the population are blasts). Granulocytic cell differentiation occurred in a similar extent in the cases of NB4 and HL60 cells and in even higher extent in the case of APL patient cells after treatment with our proposed new combination compared to conventional treatment. It means that even though more cells entered apoptosis after enhanced treatment, this did not impair differentiation efficiency.

Also, we evaluated differentiation associated* CEBPE*,* PPARG*,* CSF3R*, and* CSF3* genes expression changes after NB4, HL60, and APL patient blasts' treatment with different combinations of 3-Deazaneplanocin A, Belinostat, Idarubicin, and Retinoic acid for 6 hours and for 72 hours (Figure 3(b)). Interestingly, differentiation associated gene expression response to the treatment was different in APL patient cells compared to NB4 and HL60 cells. All tested genes' expression significantly elevated after NB4 and HL60 cell treatment, while APL patient cells treatment caused elevation of only* CEBPE* gene, but* CSF3R*,* CSF3* gene expression significantly decreased. Thus, enhanced treatment did not impair differentiation efficiency but caused different molecular response in NB4 and APL patient blasts.

### 3.4. Enhanced Treatment Caused Epigenetic Remodelling

**Figure 4:**
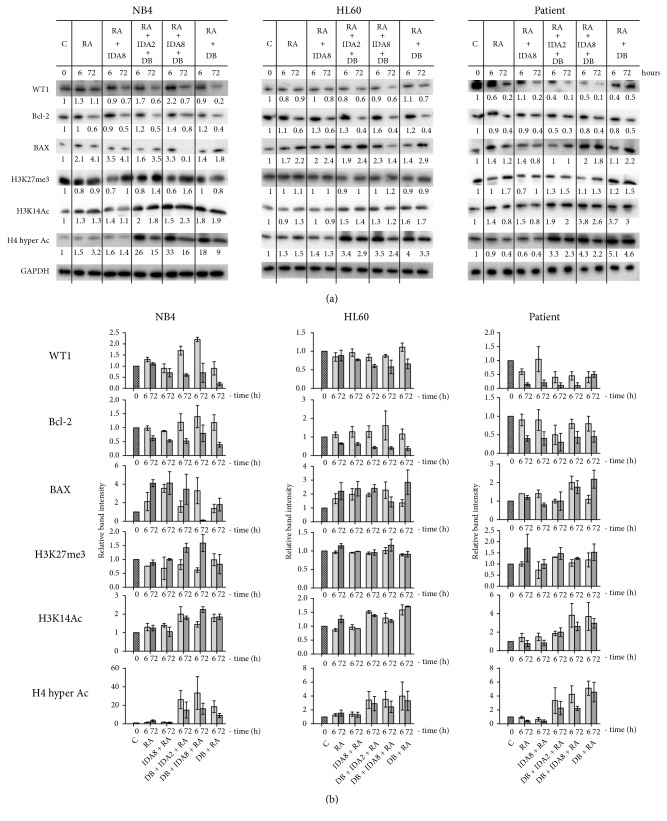
*Protein expression changes after NB4, HL60, and APL patient's cell treatment with epigenetic agents in combination with conventional treatment*. Cell samples were treated with 1 *μ*M Retinoic acid (RA), 2 nM or 8 nM Idarubicin (IDA2 or IDA8), 0.5 *μ*M 3-Deazaneplanocin A (D), and 0.2 *μ*M Belinostat (B) in different combinations for 6 and 72 hours; C: untreated cells. (a) Protein level changes were assessed by immunoblot analysis. Intensity of protein bands was measured using ImageJ software and normalized to the GAPDH loading control; results are presented as changes in comparison to untreated cells. (b) Graphical visualization of relative band intensity of detected protein levels as measured using ImageJ software and normalized to the GAPDH loading control; results are mean ± S.D. (n=2).

Since 3-Deazaneplanocin A and Belinostat are epigenetic modifiers, we evaluated their potency to induce epigenetic changes; we tested H3K27 methylation, H3K14, and H4 acetylation level changes after NB4, HL60, and APL patient cell treatment with different combinations of 3-Deazaneplanocin A, Belinostat, Idarubicin, and Retinoic acid (Figure 4). Although we did not detect the decrease in H3K27me3 level, we observed significantly increased acetylation of histones H3K14 and H4 after treatment with agent combinations containing 3-Deazaneplanocin A and Belinostat.

### 3.5. Enhanced Treatment Decreased Oncogene Expression

**Figure 5:**
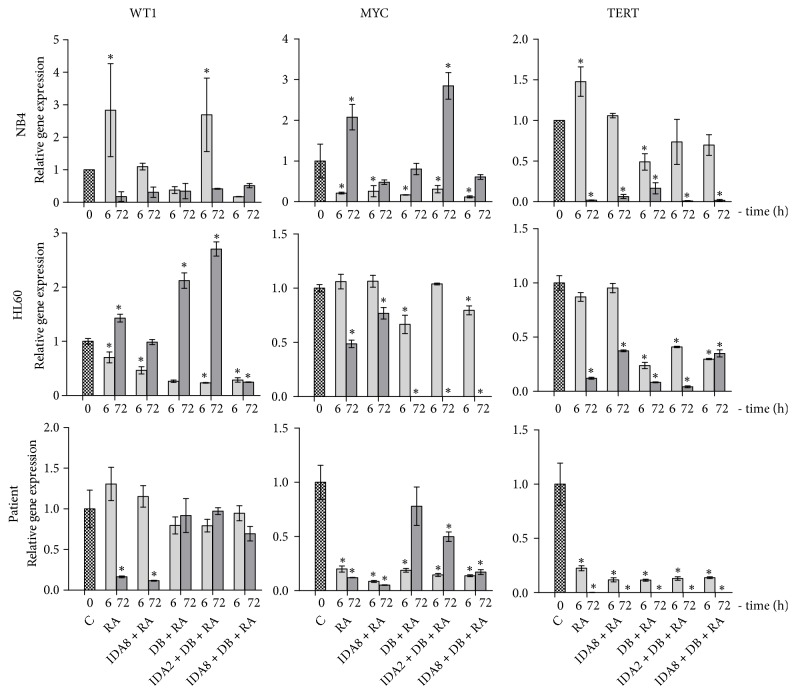
*Evaluation of oncogene expression changes after NB4, HL60, and APL patient's cell treatment with epigenetic agents in combination with conventional treatment*. Cell samples were treated with 1 *μ*M Retinoic acid (RA), 2 nM or 8 nM Idarubicin (IDA2 or IDA8), 0.5 *μ*M 3-Deazaneplanocin A (D), and 0.2 *μ*M Belinostat (B) in different combinations for 6 and 72 hours; C: untreated cells. Oncogene expression changes after treatment were measured using RT-qPCR ΔΔCt method. GAPDH was used as a “housekeeping” gene; results are presented as changes in comparison to untreated cells; results are mean ± S.D. (n = 3); *∗*P ≤ 0.05, calculated by one-way ANOVA statistical test.

In this study, we analysed oncogenes* WT1*,* MYC*, and* TERT* relative gene expression after 6 and 72 hours of treatment. All tested oncogenes' expression decreased in NB4, HL60, and APL patient cells, as assessed using RT-qPCR method (Figure 5). We further demonstrated that WT1 protein level also decreased after cell treatment (Figure 4), which confirmed* WT1* gene downregulation. Conventional treatment alone (Idarubicin + Retinoic acid) was sufficient for oncogene downregulation: enhancement with epigenetic agents 3-Deazaneplanocin A and Belinostat did not increase the effect. On the other hand, it is beneficial to elucidate that epigenetic remodelling by HMT and HDAC inhibitors did not deregulate tested oncogene expression.

## 4. Discussion

In the study, we investigated the potential of conventional treatment (Idarubicin + Retinoic acid) that enhanced with epigenetic agents to treat APL. We tested HMT inhibitor 3-Deazaneplanocin A and HDAC inhibitor Belinostat in combination with Idarubicin and Retinoic acid* in vitro* (NB4 cell line) and* ex vivo* (APL patient bone marrow cell samples). HDAC inhibitors are widely researched to treat various disorders such as cancer, neurodegenerative disorders, and immune disorders [18]. Some of them are already approved as drugs (Vorinostat, Belinostat, Panobinostat, and Romidepsin) [19]. In general, HDAC inhibitors were shown to inhibit cancer cell growth, cause apoptosis, and induce cell differentiation [19]. It was demonstrated that low acetylation levels correlate with negative outcomes [20]. HMT enzymes have not been studied as extensively; however, their deregulation was recognized as a hallmark of cancer. It has been demonstrated that reversible histone lysine methylation is involved in cell proliferation, differentiation, DNA repair, and recombination [21]. Thus, histone methyltransferases are also perspective drug targets and their inhibitors are an interesting approach for leukemia treatment [22].

Combinations of various therapies usually have synergistic or additive effects. Thus, epigenetic agents tend to be combined with other therapeutics [19]. It was demonstrated that the combination of 3-Deazaneplanocin A (HMT inhibitor) and Vorinostat (HDAC inhibitor) had synergistic effect on non-small-cell lung carcinoma cells, which might be explained by the fact that HDAC activity is required for histone methyltransferase EZH2 caused transcriptional repression (EZH2 interacts with HDACs through PRC2 protein EED) [23]. Cotreatment with 3-Deazaneplanocin A and Panobinostat (HDAC inhibitor) was also more effective for acute myeloid leukemia treatment in mice models [24]. We have previously demonstrated additive effect of 3-Deazaneplanocin A and Belinostat on APL treatment with Retinoic acid* in vitro* and* in vivo* [13, 14]. Here, we revealed that conventional treatment (Retinoic acid + Idarubicin) enhancement with epigenetic agents triggered chromatin remodelling (significantly increased acetylation of histones H3K14 and H4). Aberrant HDAC expression, which causes histone deacetylation, was observed in various cancer types. As HDACs are involved in numerous cancer development important processes (such as apoptosis, senescence, differentiation, and angiogenesis), their inhibition is an attractive therapeutic approach [25]. In our study, significantly increased histone acetylation after combined treatment shows that the treatment likely caused enhanced gene transcription coherent to cancer cell proliferation inhibition and induction of differentiation.

Moreover, we demonstrated that 3-Deazaneplanocin A and Belinostat enhanced conventional treatment (Idarubicin + Retinoic acid), causing inhibition of APL cell proliferation and survival. Cytotoxicity did not increase highly. Staining with Annexin V / Propidium Iodide and detected downregulation of antiapoptotic protein Bcl-2, upregulation of proapoptotic protein BAX, and activation of Caspase-3 showed that our proposed new combination intensified cell apoptosis. Also, treatment with 3-Deazaneplanocin A, Belinostat, Retinoic acid, and Idarubicin combination had the highest effect on metabolic activity impairment. It has been demonstrated by many scientists before that 3-Deazaneplanocin A inhibited proliferation and caused apoptosis in various cancer cells. For example, one study showed that 3-Deazaneplanocin A inhibited growth, induced apoptosis, caused senescence, and changed cell cycle related protein expression in colon cancer cells [26]. Another study demonstrated that 3-Deazaneplanocin A blocked malignant peripheral nerve sheath tumour cell growth and survival in mouse xenograft models* in vivo* [27]. Moreover, 3-Deazaneplanocin A was shown not to alter mice behaviour and of all tested organs it had irreversible side effects only on testis (caused effects are commonly found in most chemotherapy treatments) [28]. Similarly, Belinostat inhibited cell growth and induced apoptosis in various human cells and in APL cell lines NB4 and HL60 [15]. Belinostat is already approved as a drug; it is safe and is generally well tolerated [29]. To conclude, our proposed combination (3-Deazaneplanocin A, Belinostat, Retinoic acid, and Idarubicin) had higher effect on inhibition of tested cell proliferation and survival and on induction of apoptosis than conventional treatment alone (Retinoic acid + Idarubicin).

Cell cycle analysis revealed that the proposed combination caused cell cycle arrest in G0/G1 phase except that HL60 treatment with combinations with higher dose of Idarubicin (8 nM) for 24 hours caused cell cycle arrest in the phase G2. It is known that Idarubicin inhibits DNA topoisomerase II, thus disrupting DNA synthesis and arresting cells in the phase G2 [30]. Meanwhile, Retinoic acid and Belinostat caused NB4 and HL60 cell cycle arrest [15] and 3-Deazaneplanocin A caused gastric cancer cell accumulation at the phase G0/G1 [31]. Thus, the combination of 3-Deazaneplanocin A, Belinostat, and Retinoic acid with lower dose of Idarubicin (2 nM) also arrested NB4 and HL60 cell cycle at the phase G0/G1. Gene expression analysis of cell cycle inhibitors* ATM*,* p21*,* p27*, and* Rb* and cell cycle activator* CCNE2* supported these findings. Since* p53* is mutated in NB4 and HL60 cells [16, 17] and its expression did not increase in APL patient cells (Figure 2(b)), cell cycle inhibition in our tested cells might be regulated by p53-independent pathways.

We also determined that newly proposed combination accelerated granulocytic cell differentiation in higher extent compared to conventional treatment. Granulocytic differentiation results obtained by nitro blue tetrazolium (NBT) assay were supported by elevated CEBPE and PPARG gene expression. It complies with previous findings that 3-Deazaneplanocin A and Belinostat enhanced Retinoic acid induced granulocytic differentiation [13]. However,* CSF3R* and* CSF3* gene expression changes were opposite to each other: while their expression increased in NB4 and HL60 cells, it significantly decreased in APL patient blasts (Figure 3(b)).* CSF3* codes for the granulocyte colony-stimulating factor (G-CSF);* CSF3R* codes its receptor. G-CSF induces myeloid cell proliferation and survival, followed by neutrophilic differentiation [32]. The differences could be explained by high variance among blasts from different patients. For some subsets of patients, leukemic blasts proliferate spontaneously, whereas for other patients exogenous growth factors/cytokines are required for blast proliferation and differentiation [33]. APL patient cells were cultured under the same conditions as NB4 and HL60 cells (without exogenous growth factors); thus, this might explain lower proliferation and differentiation levels and the decrease of* CSF3 *and* CSF3R* gene expression in APL patient cells. Although combined treatment caused different molecular response in APL blasts as compared to tested cell lines, differentiation was induced successfully in all tested cells.

One of the reasons of blocked cell differentiation might be elevated oncogene expression, which is very common in cancer cells. For example, transcription factor MYC promotes cell survival and drug resistance [34]. Its deregulation might cause uncontrolled cell proliferation, inhibit myeloid cell differentiation, and introduce other cancerous changes.* MYC* is overexpressed in many cancer cases. Therefore, it is an attractive target for cancer therapeutics [35]. Retinoic acid was demonstrated to restrict MYC level [36]. Another oncogene,* WT1*, is also overexpressed in the majority of acute myeloid leukemia patients. Increased* WT1* levels are associated with resistance to therapy, a higher incidence of relapse, and poor overall survival [37].* TERT* codes telomerase catalytic subunit, which usually is overexpressed in hematologic malignancies. It correlates with resistance to apoptosis and senescence [38]. Thus, downregulation of these genes is important for successful treatment results. We demonstrated that expression of these oncogenes was significantly downregulated after cell treatment with Retinoic acid and Idarubicin. Although, treatment enhancement with 3-Deazaneplanocin A and Belinostat did not increase the effect, these epigenetic agents did not deregulate tested oncogene expression.

## 5. Conclusions

In this study, we demonstrated that HMT inhibitor 3-Deazaneplanocin A and HDAC inhibitor Belinostat enhanced conventional treatment (Retinoic acid + Idarubicin) for acute promyelocytic leukemia* in vitro* and* ex vivo*. Treatment enhancement with epigenetic agents caused chromatin remodelling which is associated with chromatin relaxation and enhanced transcription. Such changes might upregulate genes important for leukemia treatment. This is illustrated by our results demonstrating to a greater extent inhibited cell proliferation and survival, induced apoptosis, reduced metabolic activity, and accelerated granulocytic differentiation after treatment with 3-Deazaneplanocin A, Belinostat, Retinoic acid, and Idarubicin combination. We also revealed that different experimental models, NB4 and HL60 cell lines (*in vitro*) and APL patient blasts (*ex vivo*), displayed similar response to the treatment.

## Figures and Tables

**Table 1:** Primers used for RT-qPCR analysis.

Gene	Forward and reverse primers
*ATM*	F: CTCTGAGTGGCAGCTGGAAGA
	R: TTTAGGCTGGGATTGTTCGCT

*CCNE2*	GATGGAACTCATTATATTAAAGGCTTT
	AGGAGCATCTTTAAGAGCATCAACTT

*CEBPE*	F: CAGCCGAGGCAGCTACAATC
	R: AGCCGGTACTCAAGGCTATCT

*CSF3*	F: GCTGCTTGAGCCAACTCCATA
	R: GAACGCGGTACGACACCT C

*CSF3R*	F: CTTGTGGCCTATAACTCAGCC
	R: CCCACTCAATCACATAGCCCT

*GAPDH*	F: AGTCCCTGCCACACTCAG
	R: TACTTTATTGATGGTACATGACAAGG

*MYC*	F: AATGAAAAGGCCCCCAAGGTAGTTATCC
	R: GTCGTTTCCGCAACAAGTCCTCTTC

*p21*	F: GGCAGACCAGCATGACAGATT
	R: GCGGATTAGGGCTTCCTCT

*P27*	F: TAATTGGGGCTCCGGCTAACT
	R: TGCAGGTCGCTTCCTTATTCC

*p53*	F: TAACAGTTCCTGCATGGGCGGC
	R: AGGACAGGCACAAACACGCACC

*PPARG*	F: GCTCTAGAATGACCATGGTTGAC
	R: ATAAGGTGGAGATGCAGGCTG

*Rb*	GCAGTATGCTTCCACCAGGC
	AAGGGCTTCGAGGAATGTGAG

*TERT*	F: CGTACAGGTTTCACGCATGTG
	R: ATGACGCGCAGGAAAAATG

*WT1*	F: GGCATCTGAGACCAGTGAGAA
	R: GAGAGTCAGACTTGAAAGCAGT

## Data Availability

The data used to support the findings of this study are available from the corresponding author upon request.
