# Corrigendum to “HDAC and HMT Inhibitors in Combination with Conventional Therapy: A Novel Treatment Option for Acute Promyelocytic Leukemia”

**DOI:** 10.1155/2020/1834190

**Published:** 2020-09-04

**Authors:** Aida Vitkevičienė, Giedrė Skiauterytė, Andrius Žučenka, Mindaugas Stoškus, Eglė Gineikienė, Veronika Borutinskaitė, Laimonas Griškevičius, Rūta Navakauskienė

**Affiliations:** ^1^Department of Molecular Cell Biology, Institute of Biochemistry, Life Sciences Center, Vilnius University, Sauletekio Av. 7, LT-01257 Vilnius, Lithuania; ^2^Hematology, Oncology, and Transfusion Medicine Centre, Vilnius University Hospital Santaros Klinikos, Santariskiu Str. 2, LT-08661 Vilnius, Lithuania; ^3^Institute of Clinical Medicine, Vilnius University, M. K. Ciurlionio Str. 21, LT-03101 Vilnius, Lithuania

In the article titled “HDAC and HMT Inhibitors in Combination with Conventional Therapy: A Novel Treatment Option for Acute Promyelocytic Leukemia” [1], the last author was mistakenly linked to the second affiliation instead of the first. The corrected author list and affiliations are shown above.
